# To create or to recall? Neural mechanisms underlying the generation of creative new ideas^☆^

**DOI:** 10.1016/j.neuroimage.2013.11.021

**Published:** 2014-03

**Authors:** Mathias Benedek, Emanuel Jauk, Andreas Fink, Karl Koschutnig, Gernot Reishofer, Franz Ebner, Aljoscha C. Neubauer

**Affiliations:** a Department of Psychology, University of Graz, 8010 Graz, Austria; b Department of Radiology, Medical University of Graz, 8010 Graz, Austria

**Keywords:** Creativity, fMRI, Human cognition, Memory retrieval, Inferior parietal cortex

## Abstract

This fMRI study investigated brain activation during creative idea generation using a novel approach allowing spontaneous self-paced generation and expression of ideas. Specifically, we addressed the fundamental question of what brain processes are relevant for the generation of genuinely new creative ideas, in contrast to the mere recollection of old ideas from memory. In general, creative idea generation (i.e., divergent thinking) was associated with extended activations in the left prefrontal cortex and the right medial temporal lobe, and with deactivation of the right temporoparietal junction. The generation of new ideas, as opposed to the retrieval of old ideas, was associated with stronger activation in the left inferior parietal cortex which is known to be involved in mental simulation, imagining, and future thought. Moreover, brain activation in the orbital part of the inferior frontal gyrus was found to increase as a function of the creativity (i.e., originality and appropriateness) of ideas pointing to the role of executive processes for overcoming dominant but uncreative responses. We conclude that the process of idea generation can be generally understood as a state of focused internally-directed attention involving controlled semantic retrieval. Moreover, left inferior parietal cortex and left prefrontal regions may subserve the flexible integration of previous knowledge for the construction of new and creative ideas.

## Highlights

• Functional imaging was performed during spontaneous self-paced idea generation.
• Overt responses were recorded and evaluated for novelty and creativity.
• Brain activation of newly created and recalled ideas was compared.
• The generation of new ideas involved stronger activation of the left IPC.
• Creativity of ideas was related to activation of the left IFG.

## Introduction

The basis of all innovation is a creative idea. The neuroscientific investigation of creativity hence strives to unveil the specific neural processes leading to creative thought. Relevant research has revealed valuable insights into the brain activation related to divergent thinking by contrasting tasks involving higher and lower creative task demands (Abraham et al., 2012; Chrysikou and Thompson-Schill, 2011; Ellamil et al., 2012; Fink et al., 2007, 2009a; Vartanian et al., 2013). So far, however, research has not investigated the brain activity patterns specifically related to ideas of varying levels of quality. Specifically, the process of idea generation usually involves two types of ideas: ideas being recalled from memory and ideas newly created during the task (Gilhooly et al., 2007). The present study hence aims at determining the brain activation specifically related to generation of new and creative ideas in contrast to ideas recalled from memory during the spontaneous process of idea generation.

Over the last few years there has been an increasing interest in the investigation of the neural correlates of creativity, resulting in a considerable number of studies using a variety of tasks and neuroscientific methods. Recent efforts to integrate the available findings, however, reported difficulties in detecting consistent findings across studies, and identifying the most relevant brain areas involved in creative thought (Arden et al., 2010; Dietrich and Kanso, 2010; Fink and Benedek, 2013, in press). One assumed reason for these inconsistent findings may be related to the large variety of conceptual approaches employed in the field. Studies that investigated creativity employed divergent thinking tasks, verbal and figural insight tasks, mental imagery, or the generation of creative stories, paintings, or melodies (e.g., Aziz-Zadeh et al., 2012; Berkowitz and Ansari, 2010; Ellamil et al., 2012; Fink et al., 2009a; Goel and Vartanian, 2005; Howard-Jones et al., 2005; Jung-Beeman et al., 2004). Given this diversity of approaches it may become understandable that a variety of cognitive processes were found to be involved.

The present study focuses on divergent thinking which can be described as the process or ability to generate new and creative ideas to given open problems (Flaherty, 2005; Sternberg and Lubart, 1996). Divergent thinking ability is conceived of as a useful estimate for the potential of creative thought (Runco and Acar, 2012), and has reasonable predictive validity (Plucker, 1999). A common example task is the alternate uses task, which requires thinking about creative uses for common objects such as a car tire. The process of divergent thinking corresponds to the general concept of creative idea generation. There are many possible responses to this task and people differ in the fluency and originality/creativity of their responses (Guilford, 1950; Runco and Acar, 2012). Divergent thinking is thought to rely on cognitive processes such as “the retrieval of existing knowledge from memory and the combination of various aspects of existing knowledge into novel ideas” (Paulu and Brown, 2007, p. 252; see also, Mednick, 1962). Moreover, there is increasing evidence that the ability to generate highly creative responses is related to effective executive functions and intelligence (Beaty and Silvia, 2012; Benedek et al., 2012a; Benedek and Neubauer, 2013; Gilhooly et al., 2007; Jauk et al., 2013, in press; Nusbaum and Silvia, 2011).

The brain activation associated with divergent thinking has been examined with different methods including EEG and fMRI. Concerning EEG, there is robust evidence that divergent thinking is associated with increases in alpha band power especially at frontal sites and parietal regions of the right hemisphere (Fink and Benedek, 2013, in press). Moreover, the EEG alpha band was found to be sensitive to creativity-related demands of tasks (Fink et al., 2007; Jauk et al., 2012; Jaušovec, 1997), originality of ideas (Fink and Neubauer, 2006; Grabner et al., 2007), and to individual differences in creativity (Fink et al., 2009b; Jaušovec, 2000; Martindale and Hasenfus, 1978). Increases in alpha power presumably reflect increased internal attention demands and the prevalence of top-down control due to the intensive memory search during idea generation (Benedek et al., 2011; cf., Klimesch, 2012). Fink et al. (2009a) examined the brain activation related to a set of four divergent thinking tasks varying in the amount of creative task demands by means of EEG and fMRI. They found that divergent thinking generally involved strong BOLD increases in frontal regions of the left hemisphere including the inferior frontal gyrus, anterior cingulate and precentral gyrus corresponding to increased alpha activity in the EEG assessment. Divergent thinking with high creative task demands (i.e., finding creative alternate uses for objects) specifically involved higher activation of the left angular gyrus and lower activation of the right inferior parietal cortex as compared with a divergent thinking task involving low creative task demands (i.e., generating typical object characteristics). Subsequent studies investigated the effect of cognitive stimulation on creativity of ideas and brain activation pointing at the specific role of temporo-parietal regions for controlling attention to stimulation or memory cues (Fink et al., 2009a, 2010, 2012).

Abraham et al. (2012) also compared divergent thinking tasks with higher and lower creative demands (i.e., alternate uses task vs. object location task) and found that the former was related to stronger activations in the inferior and middle frontal gyri of the left hemisphere but also the left inferior parietal cortex. Divergent thinking was also contrasted to the convergent n-back revealing diverse differences across the brain including a higher involvement of the hippocampal formation during divergent thinking. Chrysikou and Thompson-Schill (2011) employed a figural version of the alternate uses task and compared conditions asking for common or uncommon uses in a between-subject design. Both divergent thinking conditions elicited activations of the left frontal cortex and of occipital brain regions; thinking about uncommon uses was found to lead to stronger occipital activations possibly related to cognitive strategies applied to the visually depicted object.

This study aims to address an important conceptual issue that has not been considered in the literature so far. Ideas arising during divergent thinking are usually defined as creative when they are unusual and appropriate (Runco, 2012; Sternberg and Lubart, 1996). This, however, does not necessarily imply that these ideas are the result of a genuinely creative process. A detailed analysis of the responses given in divergent thinking tasks revealed that people can retrieve a substantial amount of unusual ideas from memory without actually having created them (Gilhooly et al., 2007). For example, thinking about alternate uses for a car tire may elicit responses such as “swing” and “crash barrier”, which conform to the task instructions but which are not new to most people. The distinction between *old* and *new* ideas concerns a vital point of creative idea generation. Only new ideas are the result of a genuinely creative act in which previously unrelated frames of thought become associated in a new and meaningful way (Koestler, 1964). In contrast, old (i.e., known) ideas result from successful retrieval from long-term memory and thus do not involve a creative process. Therefore, this study aims to uncover the specific brain processes related to the generation of new and hence genuinely creative ideas. This is achieved by contrasting brain activation associated with the generation of new and old ideas in an event-related design. This study employs a novel experimental paradigm allowing self-paced generation and expression of ideas. This approach ensures a natural and valid condition for idea generation, paying tribute to the spontaneous nature of creative thought (Dietrich, 2004; Finke, 1996). Although research has not yet addressed this specific research question, one might expect stronger involvement of the medial temporal lobe during generation of old ideas, given its central role for declarative memory (e.g., Squire et al., 2004). We also aim at analyzing the brain activation related to high creativity of ideas which goes beyond novelty. Considering that the literature suggests that intelligence and executive processes play an important role for the generation of creative ideas, we expect that creativity of ideas should be related to activation in brain regions supporting executive functions (e.g., left prefrontal cortex; Barbey et al., 2012).

## Materials and methods

### Participants

The sample consisted of 35 healthy adults (24 female, 11 male; mean age: 22.7 years, age range: 18–29) after excluding seven participants; three due to technical problems with audio recording of responses, one for excessive head movements (> 1 mm), and three who did not meet the performance criterion (see below). All participants were right-handed, had normal or corrected-to-normal vision, and reported no history of CNS-affecting drugs, mental or neurological diseases. They gave written informed consent and were paid for participation in the fMRI session. The study was approved by the local ethics committee of the Medical University of Graz, Austria.

### Experimental task and procedure

**Fig. 1:**
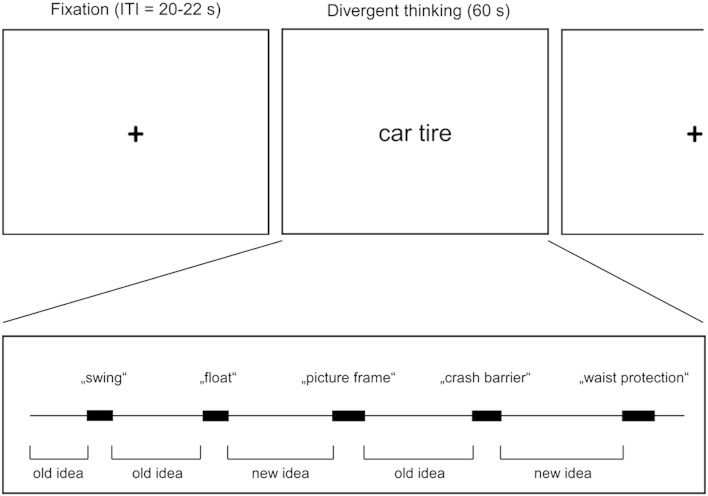
Schematic sequence of a single run. Fixation phases (20–22 s) are followed by divergent thinking phases (60 s). During divergent thinking, participants performed self-paced generation of ideas. Black boxes represent time periods in which ideas are vocalized. Idea generation epochs were modeled as time periods preceding the vocalization of ideas. Ideas were categorized as *old* or *new* after the experiment depending on whether they were retrieved from memory or newly created.

Participants performed the alternate uses task, which is a divergent thinking task that is commonly used in the behavioral and neuroscientific study of creative idea generation (Fink and Benedek, 2013, in press; Fink et al., 2007). This task requires generating creative uses for given common objects (e.g., “car tire”). Participants were asked to name all the unusual and creative uses they could think of and to vocalize their ideas as soon as they came to their mind. This mode of self-paced responding was chosen in order to capture the process of spontaneous idea generation in a natural and valid way (Birn et al., 2010; Long et al., 2010). The data was acquired in a single run consisting of 15 task blocks and 16 fixation blocks. The session started with a fixation block (25 s) followed by 15 task blocks which were separated by randomly jittered fixation null epochs (20–22 s; see Fig. 1). Each task block consisted of an idea generation period (60 s) presenting different items taken from previous studies (Fink et al., 2012). Participants' overt verbal responses were recorded by means of a funnel and a plastic tube (20 mm diameter) leading to a microphone placed outside the scanning room (Barch et al., 1999). A coworker monitored the task with headphones and immediately transcribed all responses.

The key experimental variation of this study capitalized on the fact that ideas during divergent thinking are either retrieved from long-term memory or created at that very moment (Gilhooly et al., 2007). Participants hence were asked to review all their responses right after the scanning session and to indicate for each single idea whether it represented an *old idea* or a *new idea*. This was done following a brief instruction defining an old idea as an idea that was previously known to the participant and that was remembered during the experiment (e.g., “using a car tire for building a swing”), whereas a *new idea* was previously unknown and came to mind for the first time during the experiment (e.g., “using a car tire as a picture frame”). This post hoc categorization of responses was used to assign all generated ideas to one of the two experimental conditions (i.e., old idea vs. new idea; see Fig. 1).

### Analysis of idea generation behavior

The responses of each participant were recorded automatically by the presentation software (Presentation; Neurobehavioral Systems, Albany, CA), and transcribed to a spreadsheet. Immediately after the scanning session, participants were asked to categorize the ideas generated during the fMRI session as old or new. As an additional criterion of idea quality, all responses were rated for creativity on a 4-point scale (“1, uncreative”, “2, somewhat creative”, “3, fairly creative”, and “4, very creative”) by three raters who were blind to the old/new distinction. Raters were told that creativity evaluations should reflect both originality/unusualness and appropriateness of the idea in a single holistic judgment (e.g., Benedek et al., 2013; Runco and Acar, 2012; Wilson et al., 1953), and that high creativity ratings should only be assigned to ideas that only few people could presumably come up with. The raters showed good inter-rater-reliability (ICC = .78). The ratings were used to test whether old and new ideas differ in creativity and for parametric analyses relating brain activation to creativity of ideas. Three participants who failed to generate at least 15 old and 15 new ideas in the total session were excluded from further analyses, thus keeping only participants with a reasonable number of valid events to ensure robust model estimation.

#### Response timing

The audio files of each run were used to determine the onset and durations of idea generation and speech epochs relative to stimulus onset. This was realized by means of self-devised Matlab (MathWorks, Natick, MA) software performing a voice key analysis which detects when speech rises over a given threshold for a certain length of time (e.g., Kawamoto et al., 1998). The results of this analysis were then subjected to visual inspection for final corrections.

Additionally, we analyzed whether the distribution of response latencies was adequate for fMRI analysis (Öztekin et al., 2010). *Tau*-parameters were estimated for each individual participant from the best fit of an exponential-Gaussian function to the inter-response latencies (Lacouture and Cousineau, 2008). The *tau* parameter characterizes the exponential part of the ex-Gaussian reflecting an ongoing memory search process during recall, which decays exponentially (Rohrer and Wixted, 1994). It corresponds to the lag between response events and therefore influences design efficiency. The mean *tau* in this study was 5.51 (*SD* = 2.31), which was previously associated with good design efficiency (Öztekin et al., 2010).

### fMRI procedure

Whole brain imaging was performed on a 3 T Tim Trio system (Siemens Medical Systems, Erlangen, Germany) using a 32-channel head coil. BOLD-sensitive T2*-weighted functional images were acquired using a single shot gradient-echo EPI pulse sequence (TR = 2000 ms, TE = 25 ms, flip angle = 90°, slice thickness = 3 mm, matrix size 64 × 64, FoV = 192 mm^2^, 34 slices per volume). The first two volumes after the scanner was started were discarded to allow for T1 equilibration effects. Head motion was restricted using firm padding that surrounded the head. Visual stimuli were presented using the Presentation software (Neurobehavioral Systems, Albany, CA) onto a screen and viewed through a mirror attached to the head coil.

### fMRI data analysis

Functional MRI data analysis was performed using SPM 8 software (Wellcome Department of Imaging Neuroscience, London, UK). For each participant approximately 620 functional images were obtained (variation is due to individually randomized jittering of null fixation events). Preprocessing steps included spatial realignment with unwarping (to account for movement-by-susceptibility induced variance), slice time acquisition correction, spatial normalization to an averaged EPI template in standard Montreal Neurological Institute (MNI) space (voxel size = 3 × 3 × 3 mm), and smoothing with a 6-mm full-width at half-maximum Gaussian kernel. Data were high-pass filtered (128 Hz) to account for effects of scanner drift.

Effects were estimated with a subject-specific fixed effects model including the conditions REST (i.e., fixation epochs), OLD, NEW, and SPEECH (i.e., time of overt response). Moreover, motion parameters were included in the model as regressors of no interest. The conditions OLD and NEW refer to the time periods of active idea generation and were defined as the epochs immediately before the actual vocalization of an idea, starting either at stimulus onset or after the vocalization of a previous response, respectively. These epochs were classified as either old or new depending on whether the resulting idea was retrieved from memory or newly created (for details, see above). The SPEECH condition was included to capture variance in fMRI time series related to overt responses.

The general brain activation related to divergent thinking was analyzed with the contrast of both idea generation conditions against the implicit baseline (Poline et al., 2007): OLD & NEW > 0. At the second level, a random effects analysis was performed computing one-sample t-tests for the subject-specific statistical parametric maps obtained at the first level. Voxel-based results for this general task effect are reported employing a conservative criterion, i.e., for clusters with a cluster size of k ≥ 100 significant at a level of *p* < .05 (corrected for family-wise errors; FWE).

**Fig. 2:**
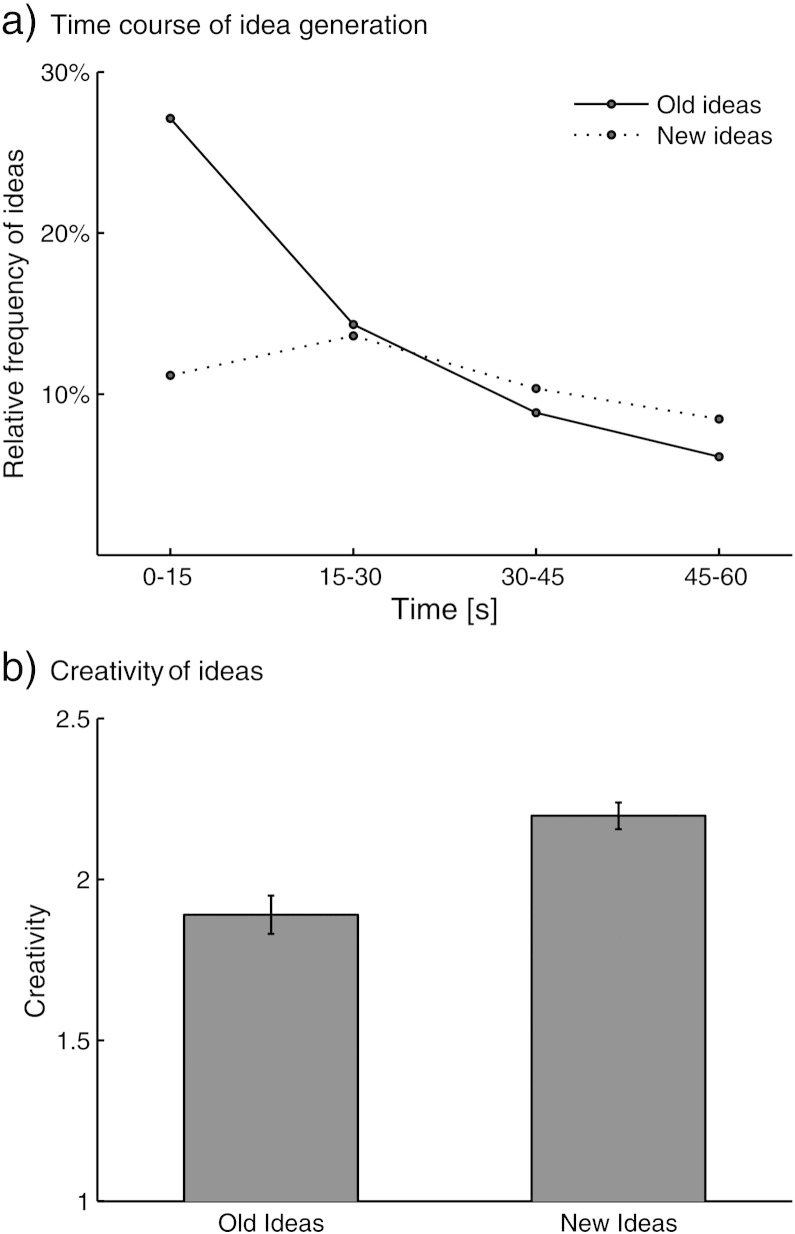
Behavioral analysis of idea generation behavior. a, Relative frequency of old and new ideas in four 15-s intervals of the divergent thinking task. b, Rated creativity of old and new ideas.

For further analyses considering brain activation at idea level, the epochs of idea generation were restricted to a constant time of 4 s directly before the idea was vocalized. The time period immediately before an idea is assumed to reflect the brain processes leading to a specific old or new idea, whereas earlier time periods may reflect more general processes involved in divergent thought (Fink et al., 2007; Jung-Beeman et al., 2004). Moreover, this procedure is essential to avoid potential biases caused by differences in response latencies between or within experimental conditions. Another potential bias in this particular context could be assumed in different production rates of old and new ideas observed within the initial 15 s (see Fig. 2). Therefore, analyses were further restricted to ideas occurring after the initial 15 s of each task block when the generation probability of old and new ideas was similar. Accordingly, idea epochs within the initial 15 s, as well as time periods preceding the 4-s pre-idea epochs, were modeled by regressors of no interest, and remaining idea epochs shorter than 4 s (12%) were excluded from the analysis. Finally, we accounted for potential effects of response length (i.e., number of letters of responses), response duration (i.e., time needed to vocalize responses), and response creativity (i.e., rated creativity of responses) by including these factors to the model as parameters at the level of single old and new idea events. Response length and response duration reflect indicators of general response complexity that were considered as regressors of no interest. Two analyses were derived from this model. First, we computed the contrast of old and new ideas (NEW > OLD), which was adjusted for differences in general response complexity and response creativity (by treating these variables as regressors of no interest). Second, we aimed at identifying brain areas that systematically increase or decrease activation as a function of the creativity of ideas. To this end, we computed the common parametric effect of response creativity for old and new ideas (OLD ∗ CREA & NEW ∗ CREA > 0). This analysis again was adjusted for parametric effects of response complexity and for any general effects related to the generation of new or old ideas. Whole brain voxel-based effects were double-thresholded and considered reliable to the extent that they were significant at voxel-level (*p* < .001) as well as at cluster-level (*p* < .05).

Signal change was computed to determine the direction (activation or deactivation) and amplitude of changes in regions showing significant condition effects using MarsBaR 0.43 (Brett et al., 2002). For computation of signal change, fixation null periods were not modeled in order to provide a well-defined baseline for activation changes. Additionally, we tested predicted differences in memory-related areas of the medial temporal lobe including left and right hippocampus and parahippocampus anatomically defined by the automated anatomically labeling (AAL) library (Tzourio-Mazoyer et al., 2002).

## Results

### Behavioral analysis of idea generation

Participants generated on average 85.37 ideas (*SD* = 31.34) in the divergent thinking tasks. An analysis of the time course of old and new ideas (the 60-s task was separated in four successive 15-s epochs) revealed that the total rate of ideas generally decreased over time, *F*(2.25, 88.38) = 83.89, *p* < .0001, partial-eta^2^ = .68. There was a higher total number of old than new ideas (*M* = 48.34 vs. 37.02 ideas; *SD* = 19.88 vs. 19.25), *F*(1,102) = 8.69, *p* = .006, partial-eta^2^ = .20. The beginning of the task was dominated by old ideas, but new ideas became more frequent at later stages, *F*(2.18, 74.22) = 51.86, *p* < .001, partial-eta^2^ = .60 (see Fig. 2a). The average response latency was shorter for old ideas than for new ideas (*M* = 6.69 vs. 8.73 s; *SD* = 2.72 vs. 3.08), *t*(34) = 6.83, *p* < .001, *d* = 1.16. When considering only ideas occurring after the initial 15 s of the task and with response latencies of more than 4 s (46.12%; see Methods section for rationale) the number of old and new ideas did not differ significantly (*M* = 18.21 vs. 21.09 ideas; *SD* = 7.30 vs. 8.02), *t*(34) = 1.48, *p* = .15, but the average response latency of old ideas was still slightly shorter than that of new ideas (*M* = 10.44 vs. 11.34; *SD* = 3.53 vs. 3.22), *t*(34) = 2.26, *p* = .03, *d* = 0.04. An analysis of the rated creativity of old and new ideas showed that new ideas were rated significantly more creative than old ideas, *t*(34) = 8.66, *p* < .0001, *d* = 1.46 (see Fig. 2b). Old and new ideas did, however, not differ significantly with respect to response length (*M* = 16.62. vs. 17.32 letters, *SD* = 4.88 vs. 5.68), *t*(34) = 1.57, *p* = .13, or response duration (*M* = 1.67 vs. 1.70 s; *SD* = 0.53 vs. 0.59), *t*(34) = 0.68, *p* = .50.

### fMRI analysis

#### Brain activation related to divergent thinking

**Fig. 3:**
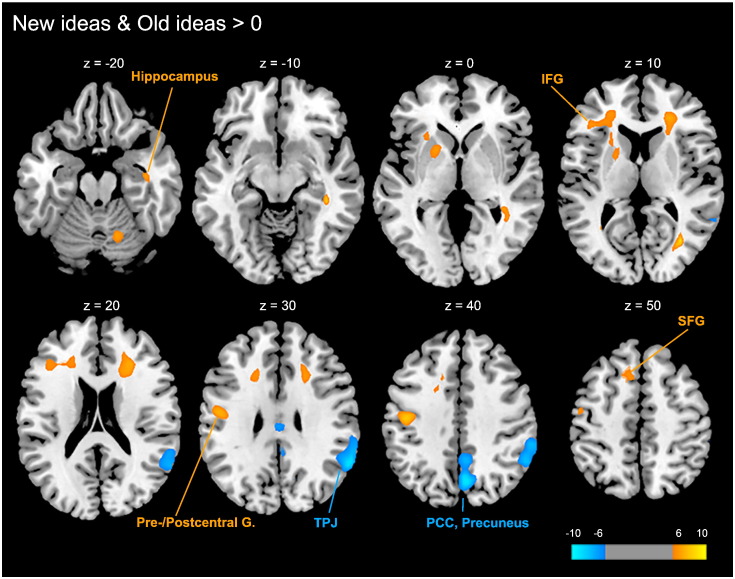
Whole brain analysis (T maps) of brain activation during divergent thinking relative to implicit baseline (OLD & NEW > 0; *p* < .05, FWE corrected, k ≥ 100). Axial planes are depicted at z = − 20 to 50. Divergent thinking is associated with significant activation in the left inferior gyrus (IFG), left superior frontal gyrus (SFG), left pre- and postcentral gyri, and right hippocampus (yellow colors), and with significant relative deactivations (blue colors) in the right temporoparietal junction (TPJ), right precuneus, and posterior cingulate cortex (PCC).

Divergent thinking (OLD & NEW > 0) was related to activation of a left-hemispheric frontal network, involving the left inferior frontal gyrus (IFG) and superior frontal gyrus (SFG) extending medially to the left insula and putamen (BA 46, 47), and of a cluster encompassing parts of the hippocampus and inferior temporal gyrus in the right hemisphere (BA 20). Further relevant regions of activation included the left precentral and postcentral gyri (BA 3,4,6), subgyral regions of the right frontal cortex and a cluster in the right posterior cerebellum (see Table 1, and Fig. 3). The reverse contrast (OLD & NEW < 0) revealed lower relative brain activation in the right temporoparietal junction (TPJ; BA 40) including the supramarginal gyrus, the angular gyrus, and posterior parts of the right superior and middle temporal gyri, as well as right-lateralized deactivations in the precuneus (BA 7) and the posterior and middle cingulate cortices (BA 31; see Table 1, and Fig. 3).

#### Brain activation related to generation of new vs. old ideas

**Fig. 4:**
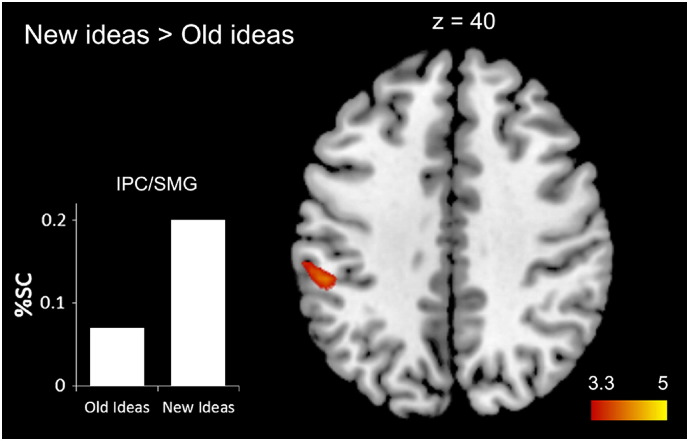
Top: Whole brain analysis (T maps) for the contrast of new vs. old ideas (double-thresholded with *p* < .001 at voxel-level and *p* < .05 at cluster-level) including %SC in the significant cluster. The generation of new ideas was associated with stronger activation in the left inferior parietal cortex (IPC) including parts of the supramarginal gyrus (SMG).

Contrasting the experimental conditions (NEW > OLD) revealed that the creation of new ideas was associated with significantly stronger brain activation in the left inferior parietal cortex (IPC; BA 40) including parts of the supramarginal gyrus (SMG; peak activation at x, y, z = − 51, − 34, 40, *t* = 4.24; k = 29; see Fig. 4). This effect is due to activation increases in the left IPC relative to baseline which is stronger during the generation of new ideas (%SC = 0.20 vs. 0.07, for new and old ideas, respectively). In contrast, the retrieval of old ideas from memory (OLD > NEW) was not associated with any significantly stronger activations.

To examine whether these effects are specific for the pre-idea time window, we also compared conditions for time periods preceding the 4-s pre-idea period. In these earlier epochs of divergent thinking, %SC in the three clusters did not differ between generation of new and old ideas (left IPC: *t*[35] = 0.49, *p* = .50). SC analyses in a priori defined regions generally revealed activation increases relative to baseline but no significant differences between conditions (left hippocampus: %SC = 0.14 vs. 0.14, *SD* = 0.31 vs. 0.32, *t*[34] = 0.11, *p* = .91; right hippocampus: %SC = 0.16 vs. 0.12, *SD* = 0.26 vs. 0.31, *t*[34] = 0.99, *p* = .33; left parahippocampus: %SC = 0.02 vs. 0.06, *SD* = 0.36 vs. 0.38, *t*[34] = − 1.07, *p* = .30; right parahippocampus: %SC = 0.13 vs. 0.12, *SD* = 0.37 vs. 0.39, *t*[34] = 0.35, *p* = .73, for new and old ideas respectively).

#### Brain activation as a function of idea creativity

**Fig. 5:**
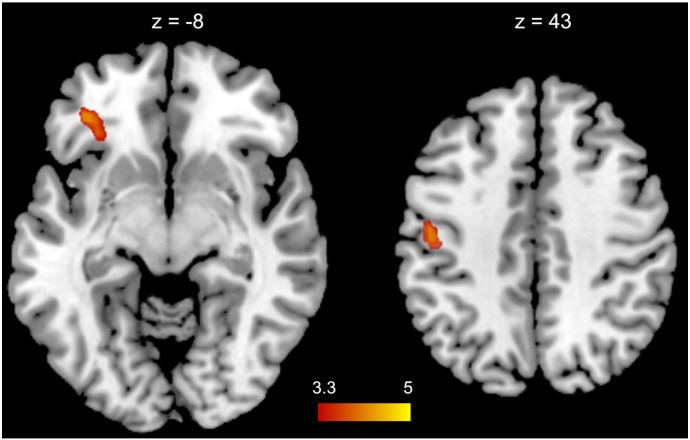
Top: Whole brain parametric analysis (T maps) for creativity of ideas (double-thresholded with *p* < .001 at voxel-level and *p* < .05 at cluster-level). Higher creativity of ideas was related to stronger brain activation in the orbital part of the left inferior frontal gyrus (IFG; depicted at z = − 8) and in a cluster located in the left precentral and postcentral gyri (depicted at z = 43).

A whole brain parametric analysis for creativity of ideas revealed that the generation of more creative ideas was related to stronger brain activation in the orbital part of the left inferior cortex (IFG; BA 47; peak activation at x, y, z = − 36, 35, − 8, *t* = 4.39; k = 22), and in a cluster located in the precentral and postcentral gyri (BA 3,4; peak activation at x, y, z = − 48, − 16, 43, *t* = 4.23; k = 38). No brain areas were found to decrease activation as a function of creativity of ideas (see Fig. 5).

## Discussion

Behavioral results revealed that divergent thinking is initially dominated by the retrieval of common, known ideas, whereas new and more creative ideas occur more frequently at later stages in the ideation process. This conforms well to psychometric findings showing that novelty and creativity of ideas generally increase over time (Beaty and Silvia, 2012; Benedek and Neubauer, 2013; Gilhooly et al., 2007). Common ideas are more accessible and thus they are generated earlier. After these dominant ideas are overcome, executive processes and cognitive strategies support the generation of new and more creative ideas (Beaty and Silvia, 2012; Benedek et al., 2012a).

### Divergent thinking effects

Divergent thinking was associated with activation of the left inferior frontal gyrus (IFG) and regions of the superior frontal gyrus (SFG), thus including regions of the left ventrolateral and dorsolateral prefrontal cortices. These regions were shown to be consistently involved in divergent thinking in previous research (e.g., Abraham et al., 2012; Fink et al., 2009a; Vartanian et al., 2013). The left IFG is known to be responsible for controlled semantic processing including retrieval and selection of semantic concepts (Badre and Wagner, 2007; Badre et al., 2005; Blumenfeld and Ranganath, 2007). The controlled retrieval, selection, and integration of stored knowledge are considered as central cognitive processes in divergent thinking which requires retrieving and selecting relevant remote associations, integration of loosely related semantic concepts, and eventually verbal elaboration of ideas (Benedek et al., 2012b; Benedek and Neubauer, 2013; Mednick, 1962; Paulus and Brown, 2007). Further relevant activations were found in areas of the medial temporal lobe (MTL) including the hippocampal regions, inferior temporal gyrus and subgyral regions of the superior temporal gyrus (STG). These structures are essential for declarative memory supporting the capacity to recollect facts and events (Binder et al., 2009; Squire et al., 2004). Finally, there were strong bilateral activations in the medial part of the precentral gyrus, which can probably be attributed to subtle preparatory motor processes during silent preparation of overt responses (Indefrey and Levelt, 2004; Shuster and Lemieux, 2005).

Divergent thinking was also related to decreased brain activation in the right temporoparietal junction (TPJ), and in a cluster involving the precuneus and the posterior cingulate gyrus. The right TPJ is considered as being a core region of the right-lateralized ventral attention network. Sustained deactivation of the ventral attention network indicates focused attention which helps to prevent reorienting to distracting bottom-up stimuli during divergent thought (Berkowitz and Ansari, 2010; Corbetta and Shulman, 2002; Corbetta et al., 2008). This finding is nicely in line with recent reports of EEG alpha synchronization over the right parietal cortex that was consistently observed during different types of divergent thinking tasks (Benedek et al., 2011; Fink and Benedek, 2013, in press). Task-related deactivation of the precuneus and the posterior cingulate gyrus, which are the components of the brain's default mode network (Anticevic et al., 2012; Fox and Raichle, 2007; Gusnard and Raichle, 2001), may indicate general goal-directed processes induced by the task. This general activation pattern is largely in line with previous studies of divergent thinking using the alternate uses task (Abraham et al., 2012; Fink et al., 2009a, 2013b; Kröger et al., 2012).

### The generation of new vs. old ideas

The generation of new ideas as compared with old ideas resulted in higher activation in the anterior part of the left inferior parietal cortex including parts of the left supramarginal gyrus (SMG). This finding is consistent with the study by Fink et al. (2010), who reported stronger activation exclusively in the left SMG during idea generation in the alternate uses as compared to the object characteristics task. The latter task requires generating typical characteristics of common objects, which can be assumed to elicit predominantly known or old responses. Contrasting this task with the alternate uses task that explicitly asks for creative responses hence may also reveal brain regions that are sensitive to the novelty of ideas. Interestingly, a different study also reported associations of creative idea generation with brain activation in the left angular gyrus (AG) located just posterior to the SMG (e.g., Fink et al., 2009a). Future research thus is challenged to unveil potentially discriminant roles of the left SMG and left AG in creative idea generation.

The inferior parietal cortex is assumed to direct attention to internal knowledge representations and was found to be especially sensitive to discriminating novel and familiar information in retrieval studies (e.g., Kahn et al., 2004; Shannon and Buckner, 2004). It is consistently involved in episodic memory retrieval, together with the medial temporal lobe and prefrontal brain regions (e.g., Cabeza et al., 2008; Wagner et al., 2005). Episodic memory reflects past personal experiences which is the kind of knowledge that is commonly used to imagine and simulate possible future events (Schacter et al., 2007, 2012). The inferior parietal cortex is also thought to be part of a core brain system that “functions adaptively to integrate information about relationships and associations from past experiences, in order to construct mental simulations about possible future events” (Schacter et al., 2007; p. 660). This definition is actually very close to common definitions of creative thought which also highlight that knowledge has to be recombined adequately to create something new and useful (Koestler, 1964; Mednick, 1962). Imagining the future and divergent thinking hence shares the common process of imagination that is needed to construct new realities, be it future events or creative new uses for objects.

We had hypothesized that the generation of old ideas might elicit stronger activation of brain regions related to declarative memory retrieval such as areas of the medial temporal lobe (Squire et al., 2004). Right hippocampal activation was generally observed during divergent thinking, and positive signal changes in MTL regions point to an involvement of declarative memory retrieval during the generation of both new and old ideas. We observed, however, no activation differences in MTL regions suggesting that the generation of new and old ideas involves the retrieval from memory to a similar extent. This is generally in line with the conception that the generation of new ideas requires continuous access to long-term memory to access various semantic concepts than can be associated in a new and creative way (Benedek et al., 2012b; Mednick, 1962). Taken together, the findings suggest that the generation of new and old ideas does not generally differ in the retrieval from declarative memory but rather more specifically differs in the involvement of episodic memory retrieval. As previously argued for the generation of future thought (e.g., Hassabis and Maguire, 2007 M; Schacter et al., 2007), recall from episodic memory is conceived as a central cognitive component for constructive processes such as imagination and thus may be equally crucial for the generation of novel ideas.

### Parametric effects of idea creativity

Parametric analyses revealed that creativity of ideas was linearly related to the brain activation level in the orbital part of the left inferior frontal gyrus. The left orbital inferior frontal cortex is consistently associated with executive functions such as prepotent response inhibition according to functional imaging studies as well as lesion studies (Barbey et al., 2012; Roberts and Wallis, 2000; Swick et al., 2008), although there is also evidence for the involvement of the right inferior frontal cortex (e.g., Aron et al., 2004). Activation in the left anterior inferior frontal gyrus was also shown to be increased when executive demands are highest during semantic retrieval (Whithney et al., 2011). In contrast, frontal lobe deficits are consistently related to mental inflexibility or to perseveration and rigidity of thought (e.g., Flaherty, 2005). This finding hence is consistent with the notion that creativity of ideas is related to brain activation in regions supporting executive control. It should be noted though that this study did not directly assess the involvement of executive processes. Recent behavioral research, however, revealed that executive processes and intelligence play an important role for the ability to come up with highly creative ideas (Beaty and Silvia, 2012; Benedek et al., 2012a; Gilhooly et al., 2007; Jauk et al., 2013, in press; Nusbaum and Silvia, 2011). Divergent thinking involves continuous selective retrieval of relevant semantic information. In this process, the generation of highly creative ideas may rely on the effective inhibition of prepotent but uncreative response alternatives thus avoiding interference and allowing access to more remotely associated concepts (e.g. Benedek and Neubauer, 2013; Gilhooly et al., 2007). Moreover, it was also suggested that the maintenance of focused attention on the task and the use of effective strategies represent further ways of executive involvement in creative idea generation (Beaty and Silvia, 2012; Benedek et al., 2011).

Significant activations related to creativity of ideas also included a region in the left precentral and postcentral gyri. The increased engagement of the left premotor areas could be speculated to reflect planning and preparation of more complex creative responses (Grèzes and Decety, 2001; Wise et al., 1999).

### Limitations

The present study employed a novel approach assessing event-related brain activation during self-paced idea generation (cf., Long et al., 2010). This design was conceived to pay tribute to the spontaneous nature of creative thought (Dietrich, 2004; Finke, 1996) and to focus on the brain processes leading to the generation of new ideas. The employed design thus serves as an alternative to the common approach of defining longer constant idea generation periods of usually about 10 to 20 s. In order to avoid biases due to variable response latencies we decided to consider a constant pre-idea period of 4 s. While this time period appears to be supported by previous literature (e.g., Fink et al., 2007; Jung-Beeman et al., 2004), it must still be considered arbitrary since there is no clear evidence on the most adequate time frame for conceiving an idea. However, we obtained some validity evidence for the specificity of the observed effects. The reported regions only differed when analyzing the 4-second period directly preceding the vocalization of an idea, but not when contrasting earlier time periods. This suggests that activations of the new–old contrast are specific to the type of the resulting idea (i.e., new or old), whereas earlier time periods may reflect more general processes of divergent thought. Moreover, supplementary analyses showed that using slightly shorter pre-idea windows did not significantly change the results.

As a second issue, the brain activation prior to vocalizing an idea was thought to reflect cognitive processes of idea generation, but it could also be assumed to merely involve the preparation or elaboration of verbal responses which could be more difficult for new ideas. We believe, however, that the reported findings are valid for the following reasons: 1) To some extent, the verbalization of an idea could be conceived to be part of the creative process. Creative ideas are required to be original and appropriate, and some authors highlight that creative ideas are often perceived as clever, thus striking people as ironic, humorous or smart (Silvia et al., 2008; Wilson et al., 1953). This implies that the actual formulation of an idea contributes to its perceived creativity, such as in poetry. 2) New and old ideas did not differ in response complexity as measured by phrase length and duration of vocalization. 3) Finally, studies comparing overt and covert response modes usually do not find stronger activations in parietal regions, but rather in temporal and inferior frontal, premotor and precentral regions (e.g., Basho et al., 2007; Shuster and Lemieux, 2005). These considerations suggest that our findings can be attributed to creative thought rather than to mere verbal preparation.

As another conceptual issue, it needs to be acknowledged that memory retrieval can be biased in many ways (Schacter and Slotnick, 2004). It is hence possible that participants sometimes retrieved a faded memory but inadvertently judged it as a novel idea rather than as an old idea. We obtained, however, evidence of the general validity of the employed procedure. First of all, new ideas were rated more creative than old ideas. Moreover, as predicted by the literature the generation of old and new followed different temporal distributions, with old ideas being more frequent in the beginning of the task and new ideas becoming more frequent at later stages of the task (see Fig. 2a).

### Conclusions

We conclude that the process of idea generation (viz., divergent thinking) can be generally understood in terms of focused, internally directed attention, and controlled retrieval. This study showed that it is possible to dissociate the brain activation related to the generation of new, creative ideas from that of old, more common ideas. The left inferior parietal cortex is particularly involved during the instantiation of novel ideas potentially effecting the flexible integration of previous knowledge for the construction and simulation of novel lines of thought. Moreover, linear increases of brain activation in orbital parts of the left inferior frontal gyrus with increased creativity of ideas may reflect an increased exertion of executive processes supporting the inhibition of dominant but uncreative ideas. These findings may help to replace the often mystified character of processes implicated in creative thought by well-established concepts of human cognition. Future challenges in this field include the reconciliation of functional findings with the emerging structural evidence on individual differences in creativity (e.g., Fink et al., 2013a; Jung et al., 2010a,b; Takeuchi et al., 2010a,b).

## Figures and Tables

**Table 1:** Whole brain analysis of the brain activation related to divergent thinking (*p* < .05, FWE corrected; k ≥ 100). Location, MNI peak coordinates, cluster size k, and maximum *t*-value of the significantly activated clusters.

Location	Peak coordinates	k	*t*-max
*OLD & NEW > 0*			
L inferior and superior frontal gyri	− 15, 20, 46	331	7.49
R subgyral region of frontal cortex	24, 35, 10	152	7.54
R hippocampus, R inferior temporal gyrus	33, − 67, 7	115	10.06
L precentral and postcentral gyri	− 45, − 16, 40	113	8.49
R cerebellum	18, − 61, − 23	105	8.18

*OLD & NEW < 0*			
R temporoparietal junction	57, − 55, 28	306	10.46
R precuneus, L/R posterior and middle cingulate cortices	6, − 67, 40	161	9.78
